# Increases in vascular nitrosylation reduce blood pressure in hypertensive rats and are associated with a decrease in angiotensin-converting enzyme tissue activity

**DOI:** 10.1007/s00210-026-05348-z

**Published:** 2026-04-23

**Authors:** Lucas C. Pinheiro, Gustavo H. Oliveira-Paula, Graziele C. Ferreira, Daniele Hummel Moreira, Jose Eduardo Tanus-Santos

**Affiliations:** 1 Department of Pharmacology, Ribeirao Preto Medical School, University of Sao Paulo, Ribeirao Preto, SP Brazil; 2 Department of Pharmacology, Federal University of Santa Catarina, UFSC, Florianópolis, SC Brazil

**Keywords:** S-nitrosoglutathione, Nitrite, S-nitrosylation, ACE, Hypertension

## Abstract

The antihypertensive effects of nitrate and nitrite are widely described. Likewise, the physiological relevance of the nitrate-nitrite-nitric Oxide (NO) cycle in cardiovascular homeostasis is well known. Nevertheless, the mechanisms underlying the effects of nitrite are not completely understood, particularly in relation to S-nitrosothiol formation. On the other hand, the angiotensin-converting enzyme (ACE) plays a central role in blood pressure regulation, and little is known about its interaction with NO. This study focused on gaining a better understanding of the impact of S-nitrosylation over blood pressure, especially on ACE. For this purpose, 2K1C hypertensive rats were treated with nitrite or S-nitrosoglutathione (GSNO), resulting in a reduction of approximately 40% in blood pressure and a significant increase in protein S-nitrosylation in the aorta, as well as in nitrosylated species in plasma. Additionally, a significant decrease in ACE activity in the aorta was observed. Treatment with buthionine sulfoximine (BSO), an inhibitor of glutathione synthesis, abolished the antihypertensive effects of nitrite and prevented the increase in protein S-nitrosylation in the aorta, which was associated with the loss of effects on ACE. These findings support the idea that an increase in S-nitrosylation in the vasculature could participate in the reduction of blood pressure and could reduce ACE activity in the vasculature.

## Introduction

The effect of oral nitrite and nitrate on blood pressure has been well established over the last decade in both humans and animals, with relevant physiological implications (Carlström et al. 2011; Ghosh et al. 2013; Pinheiro et al. 2016; Sonoda et al. 2017). Initially, the antihypertensive effects were attributed to the formation of nitric oxide (NO) from nitrite through enzymatic or non-enzymatic pathways, particularly through the reduction of nitrite to NO under hypoxic conditions mediated by deoxyhemoglobin (Lundberg et al. 1994; Cosby et al. 2003; Hendgen-Cotta et al. 2008). In parallel, nitric oxide formation from nitrite in the acidic gastric environment was demonstrated (Lundberg et al. 1994), with consequent effects on blood pressure in both experimental models and humans.(Pinheiro et al. 2012, 2015, 2018; Montenegro et al. 2017). However, given the short half-life of NO, the blood pressure–lowering effects observed for several hours cannot be solely attributed to its transient bioavailability.(Moncada et al. 1991; Kapil et al. 2010). In this context, some studies suggest that nitrosylated compounds, mainly S-nitrosothiols, and not just nitric oxide, are responsible for the long-lasting systemic effects (Maron et al. 2013; Pinheiro et al. 2015, 2016, 2018). Additionally, other research groups suggest that nitrite could directly form S-nitrosothiols in red blood cells under certain conditions (Angelo et al. 2006). Considering that S-nitrosothiols may account for the prolonged blood pressure–lowering effects of nitrite, a direct nitrosylating agent such as S-nitrosoglutathione would be expected to reduce blood pressure, in both cases through the formation of nitrosylated proteins.

Several proteins can undergo S-nitrosylation, leading to functional changes that may contribute to blood pressure reduction. Although S-nitrosothiols are known to be potent vasodilators (Choi et al. 2011; Pinheiro et al. 2015). they can also mediate transnitrosylation (Broniowska and Hogg 2012), resulting in the nitrosylation of other proteins. Protein S-nitrosylation can induce significant changes in protein function or even affect gene expression(Mengel et al. 2013). Several proteins of the renin-angiotensin system can be nitrosylated, such as PKC (Choi et al. 2011), AT1 receptor (Leclerc et al. 2006). On the other hand, the angiotensin-converting enzyme (ACE), a key target of antihypertensive drugs, has been reported to be inhibited by NO(Ackermann et al. 1998). However, no specific mechanisms have been proposed. Could S-nitrosylation be responsible for inhibiting angiotensin-converting enzyme?

Previous work from our group in hypertensive animals chronically treated with nitrite showed no reduction in plasma ACE activity, nor any change in ACE activity following in vitro exposure to nitrite (Pinheiro et al. 2014). However, plasma ACE activity is ten times lower compared to tissues such as the lung or aorta (Sharifi et al. 2003, 2004). Therefore, investigating the effects of nitrite on tissue ACE activity is relevant. Additionally, little is known about the antihypertensive mechanisms of nitrite. Given that nitrite can generate S-nitrosothiols, this study hypothesizes that S-nitrosothiols formed by nitrite or directly from GSNO could impair tissue ACE activity in 2K1C hypertensive rats.

## Methods

### Animals

This study complied with the guidelines of the Faculty of Medicine of Ribeirão Preto, University of São Paulo, and the animals were handled in accordance with the guiding principles published in the National Institutes of Health Guide for the Care and Use of Laboratory Animals. Male Wistar rats with 45–60 days, (170–180 g), obtained from the colony at the University of São Paulo, were maintained on a 12-h light/dark cycle at room temperature (22–25 °C), with free access to standard rat chow and water. Ethical committee approval number 188/2014.

### Hypertension model and assessment of blood pressure

Hypertension (two kidney, one clip; 2K1C) was induced as previously described (Rizzi et al. 2014; Pinheiro et al. 2015). Briefly, the rats were anesthetized with Ketamine (100 mg/kg) and Xylazine (10 mg/kg), the left renal artery was clipped with a silver clip (0.2 mm), whereas sham-operated rats underwent the same surgical procedure except for the clip placement. After surgery, the nonsteroidal anti-inflammatory flunixine meglumine (2.5 mg/kg, sc, Banamine; Schering Plough, Brazil) was administered.

Systolic blood pressure (SBP) was assessed weekly by tail-cuff plethysmography (Martins-Oliveira et al. 2018). To minimize the effects of stress induced by this method on blood pressure measurement, the animals were trained for one week prior to surgery.

### Experimental protocol

The animals were divided into two experimental groups. First, the effect of blood pressure reduction by nitrite or GSNO was assessed to determine whether it is associated with changes in ACE activity. A short treatment was chosen, ending in the third week after induction of 2K1C hypertension, when ACE activity and expression were increased (Ceron et al. 2012). Drug treatments were started in the four groups of animals (N = 10 rats/group) two weeks after 2K1C hypertension was induced and maintained for one week. The groups were composed by Sham (normotensive control), 2K1C control, 2K1C + nitrite (15 mg/kg, gavage) and 2K1C + GSNO (0.2 mmol/kg, gavage) The 0.2 mmol/kg dose of GSNO was used because it is equivalent in number of molecules to a dose of 15 mg/kg of sodium nitrite (Pinheiro et al. 2021). At the end of the third week, six hours after treatment, the animals are euthanized with overdoses of anesthetic and blood and aorta are collected and stored at −70 °C to further analysis. To preserve S-nitrosothiols, blood was centrifuged at 1000 g, then plasma sample was stabilized with solution of NEM (N-ethylmaleimide) (10 mmol/l) and DTPA (Diethylenetriaminepentaacetic acid)(2 mmol/L) (Feelisch et al. 2002; Pinheiro et al. 2018). Doses of 0.2 mmol/kg of nitrite or GSNO were defined due to previous studies showing a significant effect on blood pressure and an increase in total S-nitrosylation (Pinheiro et al. 2016, 2021).

The second group examined the relationship between the decrease in the availability of S-nitrosothiols and the reduction in the effect of nitrite on blood pressure, as well as its relation to ACE activity. 2K1C hypertension was induced and drug treatments were started in the six groups of animals (N = 5–8 rats/group) two weeks after 2K1C hypertension was induced and maintained for one week. The groups were composed by Sham (control normotensive), Sham buthionine sulfoximine (BSO, 1.4 mmol/kg every 12 h i.p) (Pinheiro et al. 2015). 2K1C control, 2K1C + nitrite (15 mg/kg, gavage), 2K1C + BSO and 2K1C + BSO + Nitrite (15 mg/kg, gavage). At the end of the third week, six hours after treatment the animals are euthanized like first experiment.

### Measurement of plasma nitrite and nitrosylated species concentrations

Plasma aliquots were analyzed in duplicate for their nitrite and total nitrosylated species contents using an ozone-based reductive chemiluminescence assay, as previously described (Feelisch et al. 2002; Pinheiro et al. 2018). Briefly, to measure nitrite concentrations in plasma, 50 μl of plasma samples were injected into a solution of acidified tri-iodide, purged with nitrogen in line with a gas-phase chemiluminescence NO analyzer (Sievers Model 280 NO Analyzer; Boulder, CO, USA). To measure nitroso compounds (RXNO) concentrations, 500 μl of plasma samples were treated with acid sulfanilamide (5% sulfanilamide in 1 mol/L HCl) for 5 min before injection into the solution of acidified tri-iodide, purged with nitrogen in line with the NO analyzer.

### Assessment of protein nitrosylation by resin-assisted capture (SNO-RAC) method

Total nitrosylated proteins and PKC nitrosylation were determined using the SNO-RAC (Figueiredo-Freitas et al. 2015; Pinheiro et al. 2016, 2021) 20–21 with modifications. Proteins were extracted from aortic tissue with a buffer (25 mM HEPES, 50 mM NaCl, 0.1 mM EDTA, 1% NP40 and 0.1% SDS pH 7.4) supplemented with protease inhibitor cocktail (Sigmafast^tm^ Sigma) and centrifuged at 12000 g at 4 °C for 10 min. The supernatant was added to 1.6 ml of blocking buffer (HEN Bufer: 100 mM HEPES, 1 mM EDTA and 0.1 mM neocuproine, pH 8.1 plus 2.5% SDS and 20 mM methylmethanethiosulfonate) for 20 min at 50◦C, mixing every 5 min. Then 6 ml of pre-chilled acetone was added to precipitate the proteins for 20 min at − 20◦C. The samples were centrifuged at 2000 g for 10 min at 4◦C and the pellets were washed four times with acetone 70% and suspended in 0.5 ml of HEN buffer with 1% SDS. Then the samples were incubated overnight at 4 °C with 20 mM ascorbate and 40 µl of thiopropyl-sepharose 6B under rotation. All the steps were carried out in the absence of light. The resin was washed four times with 1 ml of HEN buffer plus 1% SDS and five times with HEN buffer diluted 1:10 with 1% SDS (HEN Buffer/10 SDS) followed by elution with HEN Buffer/10 SDS plus 2% of 2-mercaptoethanol for 1 h at room temperature. To quantify the proportion of nitrosylated proteins, both, control input (i) and output (o) samples are run on a 5% SDS/PAGE gel combined with a 10% 5% SDS/PAGE gel. The run was stopped when the samples reached the 10% gel. Then the gels were stained with Coomassie Blue 0.05% and nitrosylated proteins were quantified (Amersham Image 600, GE Healtcare, Little Chalfont, Buckinghamshire, UK) using the ImageJ Program (NIH, USA). Given the low protein concentration in the output samples, we loaded two times higher protein concentration for output samples as compared to input samples. The percentage of protein S-nitrosylation was calculated as 100% × 2(i)/(o).

### ACE activity assay

Aortic ACE activity was determined by a fluorimetric assay using Hippuryl-His-Leu (Sigma) as the substrate (Pinheiro et al. 2014). Briefly, 20 μL of homogenized aorta were incubated with 210 μL of Tris buffer (Tris 20 mol/L/NaCl 0.3 mol/L, pH 8.1) containing Hip-His-Leu [1 mmol/L] at 37 °C for 20 min. The reaction was stopped by adding NaOH 0.5 mol/L (1 mL). Then, 100 μL of OPA (o-phthaldialdehyde) was addpodered and homogenized for 4 min. After the addition of HCl 6 mol/L (200 μL), the solution was centrifuged at 3,000 rpm for 5 min and the supernatant were placed in 96-well microplate and the fluorescence was detected at 365 nm excitation and 495 nm emission. Standard curves for His-Leu were used to calculate the ACE activity (nmols/min/mL).

### *In vitro* experiments of ACE activity

To test whether S-nitrosothiol could reduce ACE activity, fresh plasma samples from normotensive rats were incubated with GSNO (1 mM), GSNO + ascorbate (1 mM), or GSNO + DTT (1 mM), as well as their respective controls (GSH, ascorbate, or DTT alone). The incubation was carried out for 30 min at 37 °C, and ACE activity was determined (*n* = 4 per concentration). Positive controls were used.

### Drugs and solutions

Thiopropyl-sepharose 6B was purchased from GE Healthcare (Little Chalfont, Buckinghamshire, UK) and all other drugs and reagents were purchased from Sigma Chemical Co. (St Louis, MO, USA). All solutions were prepared immediately before use.

### Statistical analysis

The results are expressed as means ± S.E.M. Comparisons between groups were assessed using one-way or two-way analysis of variance (ANOVA), followed by Tukey's test. Statistical analyses were performed using GraphPad Prism software (GraphPad Software Inc., San Diego, CA, USA). A probability value of P < 0.05 was considered statistically significant.

## Results

### Treatment with nitrite or GSNO reduces blood pressure, ACE activity associated with increased nitrosylated proteins in 2K1C hypertension

A series of experiments was conducted to investigate the role of S-nitrosothiols in the antihypertensive effects of nitrite. First, 2K1C rats with two weeks of induced hypertension were treated with nitrite or GSNO, an S-nitrosothiol.

**Fig. 1:**
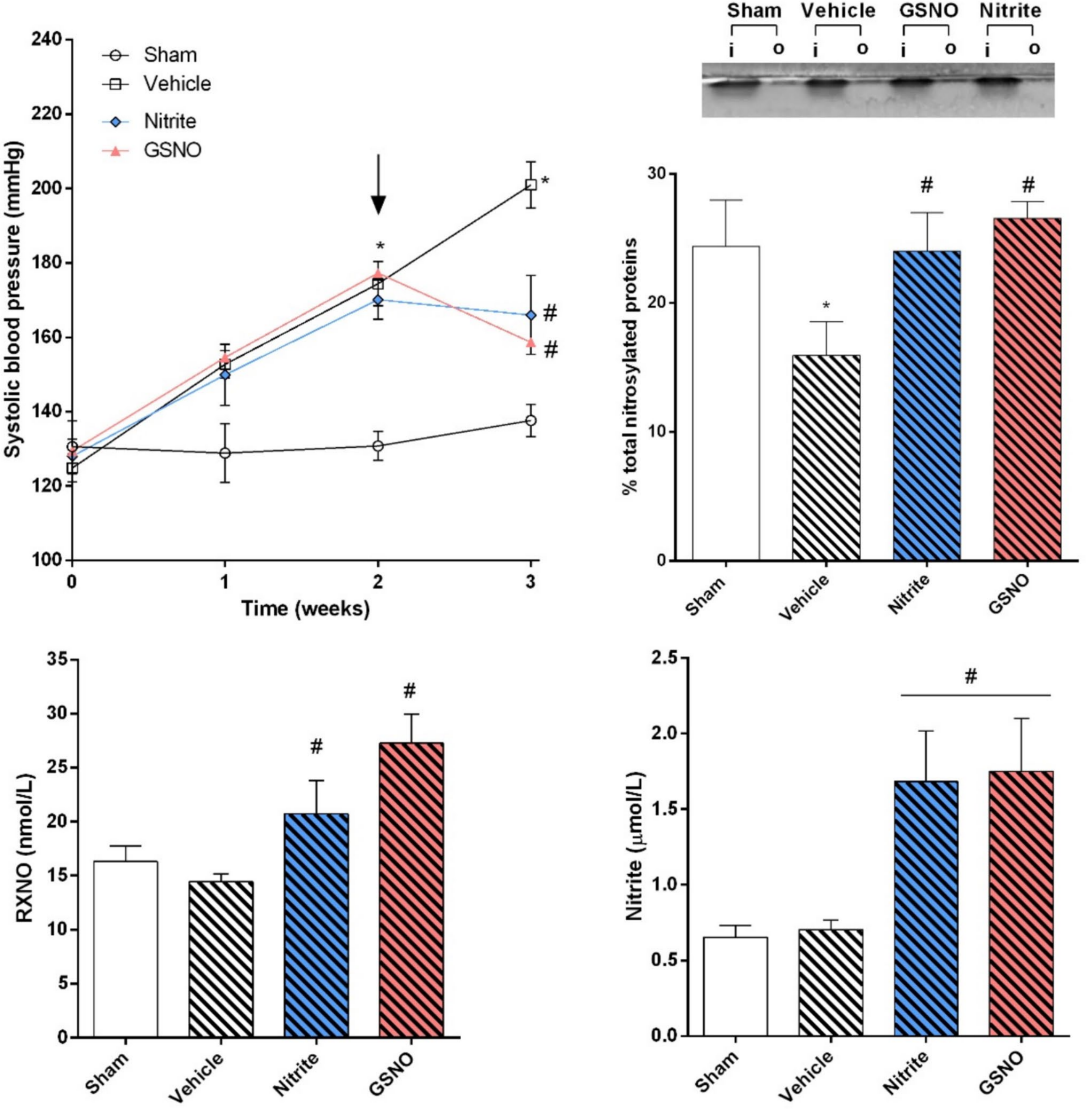
Treatment with nitrite or GSNO reduces blood pressure and increases nitrosylated proteins in early 2K1C hypertension **a**) Systolic blood pressure in 2K1C hypertensive rats treated with vehicle, nitrite or GSNO and Sham-operated rats. Treatments started after two weeks of hypertension (arrow). **b**) Total nitrosylated proteins in aorta in all groups and representative gel (i- total protein; o- nitrosylated protein). **c**) Nitrosylated species plasma in all groups **d)** Nitrite plasma levels in all groups. Data are shown as mean ± S.E.M. (n = 8–10/group in Figs* P < 0.05 *versus* Sham vehicle. # P < 0.05 *versus* 2K1C vehicle

Treatment with nitrite or GSNO reduced blood pressure within just one week of treatment (2K1C vehicle: 200 ± 6 mmHg vs. 2K1C nitrite: 166 ± 10 mmHg or 2K1C GSNO: 158 ± 7 mmHg; Fig. 1A). Hypertension was associated with a reduction in total nitrosylated proteins in the aorta (sham: 24 ± 3% vs. 2K1C vehicle: 15 ± 2%). However, both treatments increased total nitrosylated proteins in the aorta (2K1C vehicle: 15 ± 2% vs. 2K1C nitrite: 25 ± 2% or 2K1C GSNO: 27 ± 1%; P < 0.05; Fig. 1B).

Additionally, treatment with nitrite or GSNO increased total nitrosylated species (2K1C vehicle: 14 ± 1 nmol/L vs. 2K1C nitrite: 20 ± 3 nmol/L or 2K1C GSNO: 27 ± 3 nmol/L; *P* < 0.05; Fig. 1C) as well as nitrite levels in plasma (2K1C vehicle: 0.7 ± 0.1 µmol/L vs. 2K1C nitrite: 1.6 ± 0.3 µmol/L or 2K1C GSNO: 1.7 ± 0.3 µmol/L; *P* < 0.05; Fig. 1D).

**Fig. 2:**
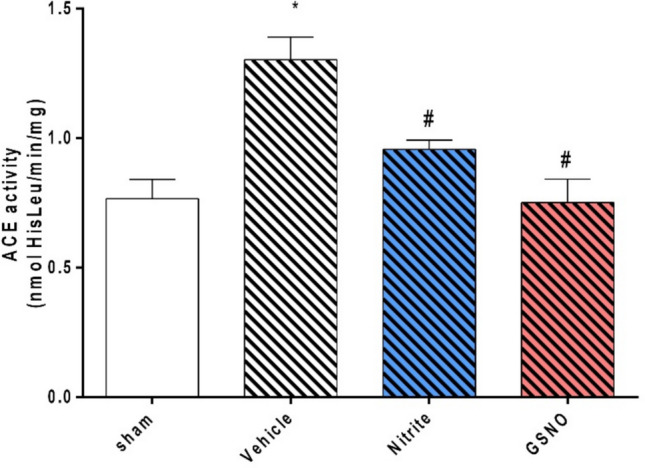
Treatment with nitrite or GSNO reduces ACE activity in early 2K1C hypertension: **a**) Angiotensin-converting enzyme (ACE) activity in aorta samples (nmol HisLeu/min/mg). *P < 0.05 versus the Sham group. #P < 0.05 versus the group of 2K1C hypertensive rats treated with vehicle. Data are shown as mean ± S.D. (*n* = 5–8 per group)

To investigate whether nitrite or GSNO treatment affects ACE activity, it was measured in the aortas of the animals. 2K1C hypertensive animals exhibited an increase in ACE activity in the aorta (Sham: 0.7 ± 0.07 vs. 2K1C vehicle: 1.3 ± 0.08 nmol His-Leu/min/mg; Fig. 2A). Both treatments reduced ACE activity compared to the hypertensive control (2K1C vehicle: 1.3 ± 0.08 vs. 2K1C nitrite: 0.9 ± 0.03 or 2K1C GSNO: 0.7 ± 0.08 nmol His-Leu/min/mg; Fig. 2).

### Concomitant treatment with BSO impairs the effects of nitrite on blood pressure reduction, ACE activity, and protein nitrosylation

**Fig. 3:**
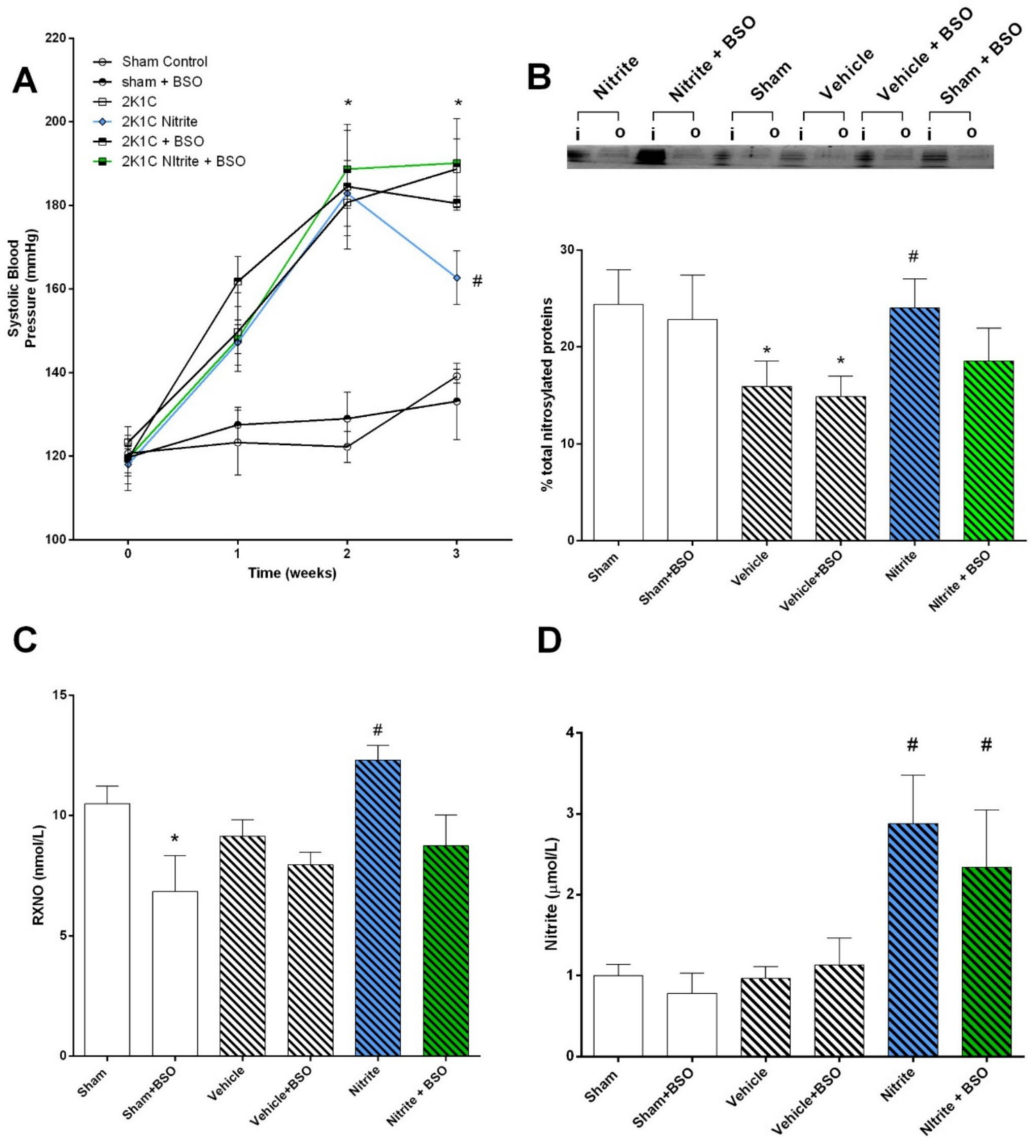
Concomitant treatment with BSO impairs nitrite effect in blood pressure and nitrosylated proteins. **a**) Systolic blood pressure in 2K1C hypertensive rats treated with vehicle, nitrite (15 mg/kg), BSO (1.4 mmol/kg) or nitrite + BSO and Sham-operated rats treated with BSO (1.4 mmol/kg) or vehicle. Treatments started after two weeks of hypertension (arrow). **b**) Total nitrosylated proteins in aorta in all groups and representative gel (i- total protein; o- nitrosylated protein). **c)** Nitrosylated species plasma in all groups **d**) Nitrite plasma levels in all groups. Data are shown as mean ± S.E.M. (*n* = 8–10/group in Figs* *P* < 0.05 versus Sham vehicle. # *P* < 0.05 versus 2K1C vehicle

**Fig. 4:**
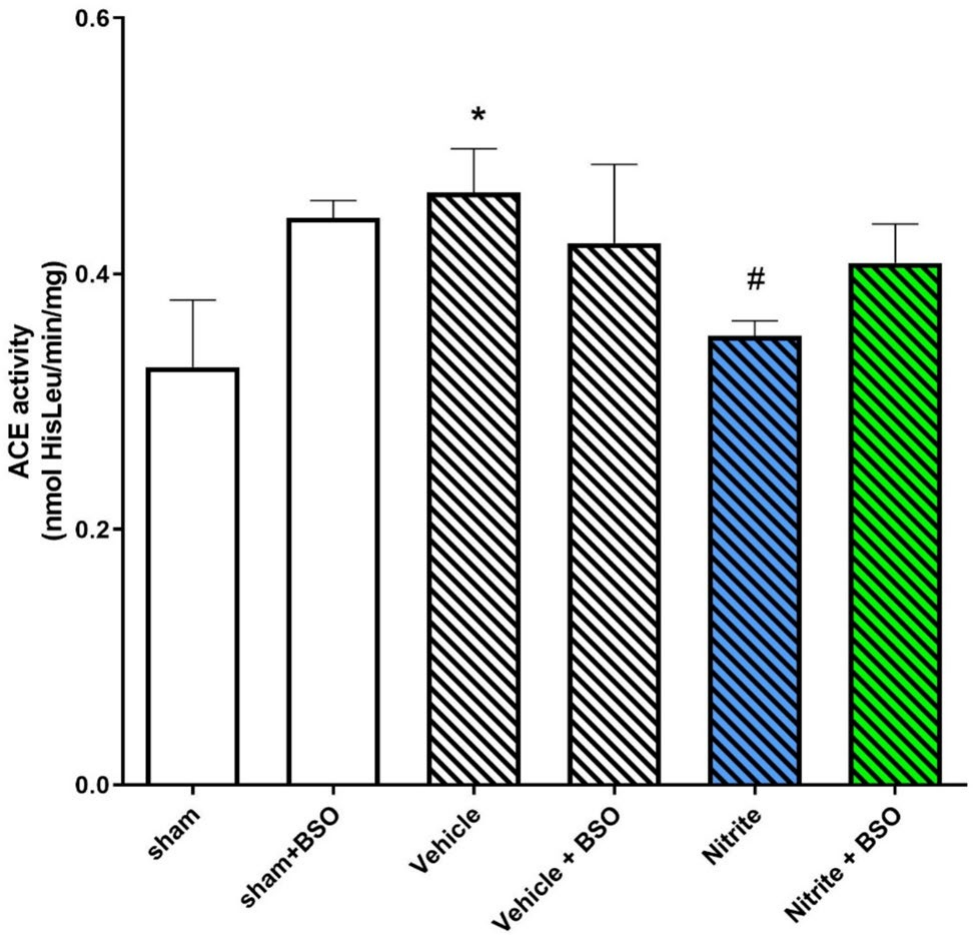
Concomitant treatment with BSO impairs nitrite reduction of activity of ACE **a)** Angiotensin-converting enzyme (ACE) activity in aorta samples (nmol HisLeu/min/mg). *P < 0.05 versus the Sham group. #*P* < 0.05 versus the group of 2K1C hypertensive rats treated with vehicle. Data are shown as mean ± S.E.M. (*n* = 5–8 per group)

To better understand the relationship between tissue nitrosylation, blood pressure, and ACE activity, 2K1C rats were treated with BSO (to reduce total glutathione levels) and nitrite, Figs. 3 and 4. Rats that received only nitrite exhibited a decrease in blood pressure, whereas co-treatment with BSO abolished the blood pressure-lowering effects of nitrite (2K1C vehicle: 188 ± 9 mmHg vs. 2K1C nitrite: 166 ± 7 mmHg or 2K1C BSO + Nitrite: 190 ± 10 mmHg; Fig. 3A). Treatment with BSO reduced the concentration of free glutathione when comparing groups that received BSO versus those that did not (Control groups 21.9 ± 10 nmol/mg protein vs. BSO groups 11.6 ± 6 nmol/mg protein, *P* < 0.05), as expected and observed in other works that used same dose of BSO (Pechanova et al. 1999; Pinheiro et al. 2015).

Additionally, BSO treatment impaired the nitrite-induced increase in total aortic nitrosylation (2K1C vehicle: 15.9 ± 2.6% vs. 2K1C nitrite: 24.0 ± 2.9% or 2K1C BSO + Nitrite: 18.0 ± 3.3%; Fig. 3B) as well as the previously observed increase in plasma nitrosylated species (2K1C vehicle: 9.1 ± 0.6 nMol/L vs. 2K1C nitrite: 12.3 ± 0.6 nMol/L or 2K1C BSO + Nitrite: 8.7 ± 1.2 nMol/L; Fig. 3C). No significant differences were observed in plasma nitrite concentrations after BSO treatment.

ACE activity in the aorta was not affected by nitrite treatment when combined with BSO, suggesting the involvement of S-nitrosothiols in the effect of nitrite on ACE (Fig. 4). However, it remains unclear whether nitrosylation directly reduces ACE activity or if the decrease in tissue expression compromises the measured activity.

### GSNO reduces ACE activity *in vitro* without nitrosylated ACE

**Fig. 5:**
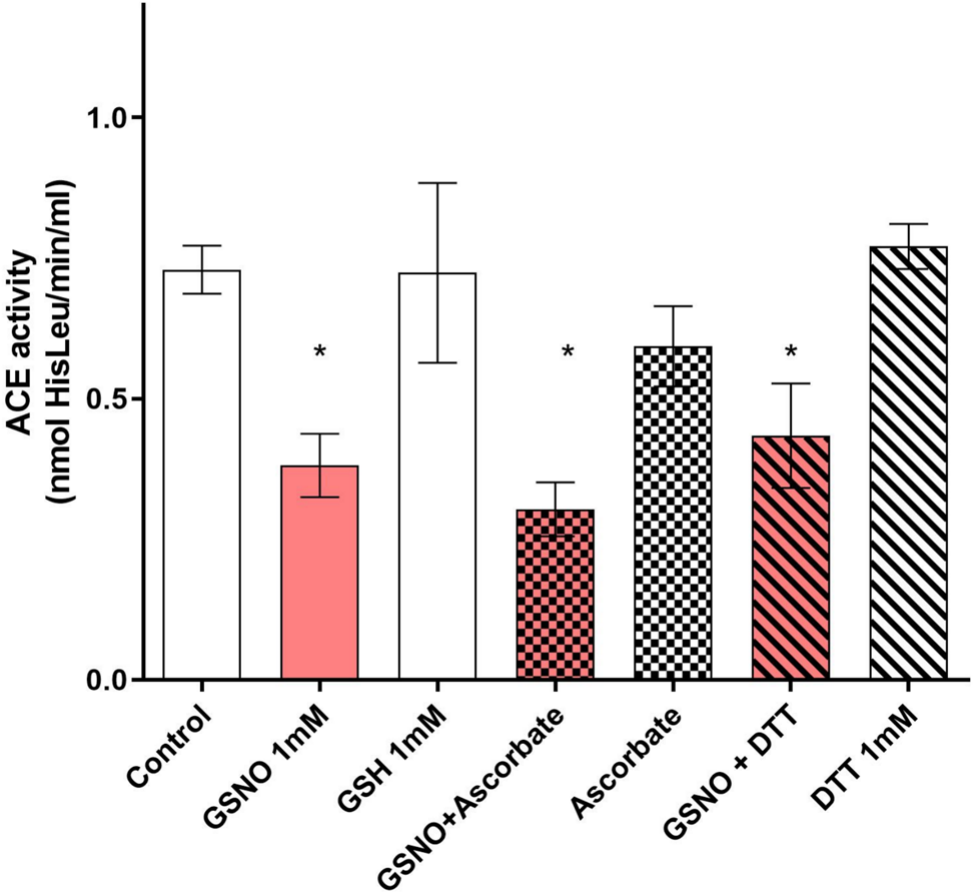
ACE activity inhibition in vitro by GSNO and Angelis Salt **a)** Angiotensin-converting enzyme (ACE) activity in plasma samples (nmol HisLeu/min/ml). ACE activity from control (plasma samples from the Sham group) treated with GSNO 1 mM, GSH 1 mM, DTT 1 mM or Ascorbate 1 mM. Data are shown as mean ± S.E.M. (n = 5 per group).*P < 0.05 versus the control

To explore the effect of nitrosylation on ACE, fresh plasma was treated with GSNO (1 mM), followed by incubation with denitrosylating agents. Exposure of plasma to GSNO (1 mM) reduced ACE activity by approximately 50% compared to the control. However, treatment with denitrosylating agents, such as ascorbate and DTT, did not reverse the effect of GSNO on ACE activity (Fig. 5). These findings suggest that the reduction in ACE activity is not due to direct nitrosylation. Additionally, none of the control treatments alone had any effect on ACE activity.

## Discussion

The main findings of this work are as follows: First, once-daily treatment with GSNO reduces blood pressure for at least 24 h. Second, treatment with nitrite or GSNO increases aortic S-nitrosylation, which is associated with decreased activity of ACE (Figs. 1 and 2). Third, the impairment of aortic S-nitrosylation through co-treatment with BSO blunts the nitrite-induced reduction of ACE activity (Figs. 3 and 4). Fourth, as previously described in the literature (Ackermann et al. 1998), NO can reduce ACE activity, but this inhibition is not due to direct nitrosylation of ACE, since denitrosylating agents are not able to reverse the NO-induced inhibition of ACE (Fig. 5).

**Fig. 6:**
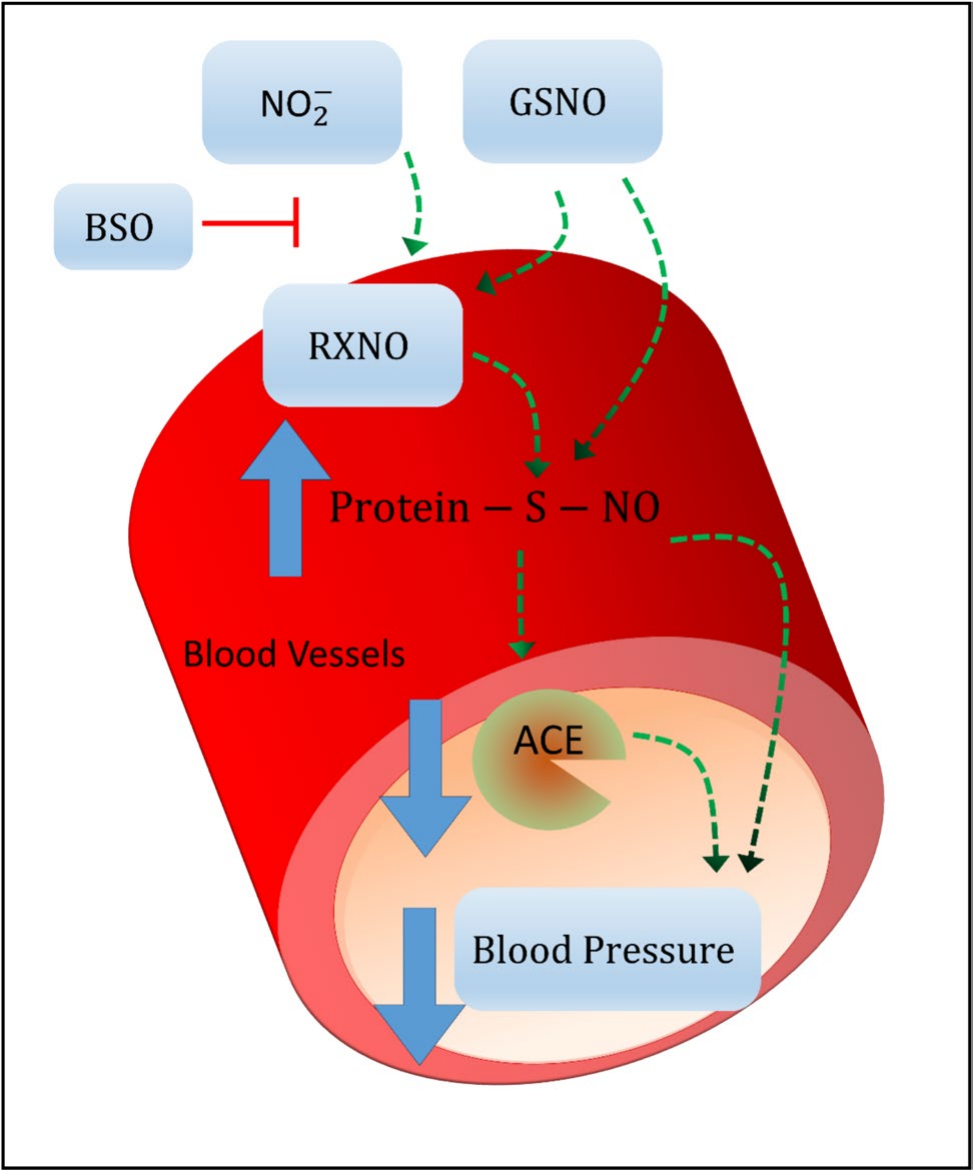
Graphical summary of the main findings of this article. Once-daily treatment with GSNO or nitrite increases vascular nitrosylation, reduces vascular ACE activity, and contributes to the decrease in blood pressure

Taken together, these data suggest that tissue S-nitrosylation—induced here by nitrite or GSNO— could be relevant to ACE activity in vascular tissue (Fig. 6). Previous studies have shown increased NO production by NO synthases in aortas subjected to shear stress, and this increase was associated with reduced ACE activity. Furthermore, during shear stress, ACE expression decreases in correlation with increased NOS activity (Rieder et al. 1997).

The vasodilatory effect of S-nitrosothiols through acute NO release is well known (Liu et al. 2016). On the other hand, the data presented here support that once-daily treatment with nitrite or GSNO can reduce blood pressure in 2K1C hypertension. This strengthens the concept that S-nitrosothiols and protein nitrosylation are central to the observed effects, as opposed to the direct action of free NO, which has a very short half-life (Moncada et al. 1991; Pinheiro et al. 2015). Moreover, concomitant treatment with BSO and nitrite leads to a decrease in protein S-nitrosylation in the aorta and in nitrosylated species in the plasma, abolishing the blood pressure-lowering effects. These findings further support the role of vascular protein nitrosylation in blood pressure regulation. In line with this, some studies have shown that increased S-nitrosylation of vascular proteins—such as PKC and angiotensin II receptors, (Leclerc et al. 2006; Choi et al. 2011; Pinheiro et al. 2021) reduces the functionality of these proteins, and consequently, the responsiveness to vasoconstrictor agents like angiotensin II or noradrenaline (Pinheiro et al. 2021; Oliveira-Paula et al. 2023). The involvement of S-nitrosylation in the antihypertensive effects of nitrite or GSNO has been investigated by several research groups (Irie et al. 2015; Jiang et al. 2016; Neto-Neves et al. 2019; Pinheiro et al. 2021). However, little is known about how changes in protein S-nitrosylation levels affect gene expression within cells.

Furthermore, the data show a decrease in blood pressure associated with reduced ACE activity in the aorta, which may be due to decreased ACE expression. However, this reduction in activity does not appear to be mediated by direct S-nitrosylation of ACE. Therefore, an increase in vascular protein S-nitrosylation could lead to reduced ACE expression and contribute to lowering blood pressure. Although the mechanisms regulating ACE expression were not fully addressed in this study, previous literature suggests that one of the main regulatory pathways involves the activation of CREB elements. These CREB elements may themselves be targets of modulation by nitrosylation (Riccio et al. 2006; Kobori et al. 2007; Nott et al. 2008). Thus, it is plausible that increased tissue nitrosylation could modulate CREB activity and, consequently, interfere with ACE expression in the aorta. Another possible pathway for the regulation of ACE expression is through NF-κB. Some studies have associated NF-κB inhibition with decreased ACE expression in tissues(Miguel-Carrasco et al. 2010). Moreover, it is well established that NF-κB can be inhibited by S-nitrosylation, and nitric oxide synthases (NOS) are a known source of NO that mediates this inhibition (Marshall and Stamler 2001; Kelleher et al. 2007). In a previous study from our group, we did not observe a decrease in plasma ACE activity; however, in the present work, we found a reduction in tissue ACE activity. This discrepancy could be explained by the fact that ACE activity in tissues such as the aorta and kidney can be up to ten times higher than in plasma (Sharifi et al. 2003). This difference in tissue activity is even more pronounced in hypertensive animals, which may explain why changes in ACE activity were not detected in plasma measurements due to their relatively low baseline activity.

Additionally, part of the effect observed in tissues could result from nitrosylated species being reduced to NO locally, thereby inhibiting ACE activity. Indeed, nitrosothiols can be reduced to NO by tissue-specific enzymes (Massa et al. 2021).

Thus, the overall data suggest a complementary mechanism of action for nitrite, involving the formation of protein S-nitrosylation and the regulation of ACE activity. Furthermore, these findings support the idea that basal concentrations of S-nitrosothiols may play a role in modulating ACE activity and expression under physiological conditions. Further studies are needed to better elucidate these questions.

## Data Availability

All source data for this work (or generated in this study) are available upon reasonable request.

## References

[CR1] Ackermann A, Fernández-Alfonso MSS, Sánchez de Rojas R et al (1998) Modulation of angiotensin-converting enzyme by nitric oxide. Br J Pharmacol 124:291–298. 10.1038/sj.bjp.0701836 9641545 10.1038/sj.bjp.0701836 PMC1565390

[CR2] Angelo M, Singel DJ, Stamler JS (2006) An S-nitrosothiol (SNO) synthase function of hemoglobin that utilizes nitrite as a substrate. Proc Natl Acad Sci U S A 103:8366–8371. 10.1073/pnas.0600942103 16717191 10.1073/pnas.0600942103 PMC1482500

[CR3] Broniowska Ka, Hogg N (2012) The chemical biology of S-nitrosothiols. Antioxid Redox Signal 17:969–980. 10.1089/ars.2012.4590 22468855 10.1089/ars.2012.4590 PMC3411335

[CR4] Carlström M, Persson AEG, Larsson E et al (2011) Dietary nitrate attenuates oxidative stress, prevents cardiac and renal injuries, and reduces blood pressure in salt-induced hypertension. Cardiovasc Res 89:574–585. 10.1093/cvr/cvq366 21097806 10.1093/cvr/cvq366

[CR5] Ceron CS, Rizzi E, Guimaraes DA et al (2012) Time course involvement of matrix metalloproteinases in the vascular alterations of renovascular hypertension. Matrix Biol 31:261–270. 10.1016/j.matbio.2012.01.009 22342460 10.1016/j.matbio.2012.01.009

[CR6] Choi H, Tostes RC, Webb RC (2011) S-nitrosylation inhibits protein kinase c-mediated contraction in mouse aorta. J Cardiovasc Pharmacol 57:65–71. 10.1097/FJC.0b013e3181fef9cb 20966762 10.1097/FJC.0b013e3181fef9cb PMC3022953

[CR7] Cosby K, Partovi KS, Crawford JH et al (2003) Nitrite reduction to nitric oxide by deoxyhemoglobin vasodilates the human circulation. Nat Med 9:1498–1505. 10.1038/nm954 14595407 10.1038/nm954

[CR8] Feelisch M, Rassaf T, Mnaimneh S et al (2002) Concomitant S‐, N‐, and heme‐nitros(yl)ation in biological tissues and fluids: implications for the fate of NO in vivo. FASEB J 16:1775–1785. 10.1096/fj.02-0363com 12409320 10.1096/fj.02-0363com

[CR9] Figueiredo-Freitas C, Dulce RA, Foster MW et al (2015) S -nitrosylation of sarcomeric proteins depresses myofilament Ca 2+ sensitivity in intact cardiomyocytes. Antioxid Redox Signal 23:1017–1034. 10.1089/ars.2015.6275 26421519 10.1089/ars.2015.6275 PMC4649751

[CR10] Ghosh SM, Kapil V, Fuentes-Calvo I et al (2013) Enhanced vasodilator activity of nitrite in hypertension. Hypertension 61:1091–1102. 10.1161/HYPERTENSIONAHA.111.00933 23589565 10.1161/HYPERTENSIONAHA.111.00933

[CR11] Hendgen-Cotta UB, Merx MW, Shiva S et al (2008) Nitrite reductase activity of myoglobin regulates respiration and cellular viability in myocardial ischemia-reperfusion injury. Proc Natl Acad Sci U S A 105:10256–10261. 10.1073/pnas.0801336105 18632562 10.1073/pnas.0801336105 PMC2481313

[CR12] Irie T, Sips PY, Kai S et al (2015) S-nitrosylation of calcium-handling proteins in cardiac adrenergic signaling and hypertrophy. Circ Res 117:793–803. 10.1161/CIRCRESAHA.115.307157 26259881 10.1161/CIRCRESAHA.115.307157 PMC4600453

[CR13] Jiang H, Polhemus DJ, Islam KN et al (2016) Nebivolol acts as a S-nitrosoglutathione reductase inhibitor: a new mechanism of action. J Cardiovasc Pharmacol Ther 21:1–8. 10.1177/1074248415626300 10.1177/1074248415626300 26746429

[CR14] Kapil V, Milsom AB, Okorie M et al (2010) Inorganic nitrate supplementation lowers blood pressure in humans. Hypertension 56:274–281. 10.1161/HYPERTENSIONAHA.110.153536 20585108 10.1161/HYPERTENSIONAHA.110.153536

[CR15] Kelleher ZT, Matsumoto A, Stamler JS, Marshall HE (2007) NOS2 regulation of NF-κB by S-nitrosylation of p65. J Biol Chem 282:30667–30672. 10.1074/jbc.M705929200 17720813 10.1074/jbc.M705929200

[CR16] Kobori H, Nangaku M, Navar LG, Nishiyama A (2007) Intratubular renin-angiotensin system: from physiology to the pahobiology of hypertension and kidney disease. Harmacological Rev 59:251–287. 10.1124/pr.59.3.3.251 10.1124/pr.59.3.3 17878513

[CR17] Leclerc PC, Lanctot PM, Auger-Messier M et al (2006) S-nitrosylation of cysteine 289 of the AT1 receptor decreases its binding affinity for angiotensin II. Br J Pharmacol 148:306–313. 10.1038/sj.bjp.0706725 16565729 10.1038/sj.bjp.0706725 PMC1751562

[CR18] Liu T, Schroeder HJ, Zhang M et al (2016) S-nitrosothiols dilate the mesenteric artery more potently than the femoral artery by a cGMP and L-type calcium channel-dependent mechanism. Nitric Oxide 58:20–27. 10.1016/j.niox.2016.05.006 27235767 10.1016/j.niox.2016.05.006 PMC6322392

[CR19] Lundberg JOM, Weitzberg E, Alving K (1994) Intragastric nitric oxide production in humans: measurements in expelled air. Gut 35:1543–1546. 10.1136/gut.35.11.1543 7828969 10.1136/gut.35.11.1543 PMC1375608

[CR20] Maron Ba, Tang S-S, Loscalzo J (2013) S -nitrosothiols and the S -nitrosoproteome of the cardiovascular system. Antioxid Redox Signal 18:270–287. 10.1089/ars.2012.4744 22770551 10.1089/ars.2012.4744 PMC3518544

[CR21] Marshall HE, Stamler JS (2001) Inhibition of NF-κB by S-nitrosylation. Biochemistry 40:1688–1693. 10.1021/bi002239y 11327828 10.1021/bi002239y

[CR22] Martins-Oliveira A, Guimaraes DA, Ceron CS et al (2018) Direct renin inhibition is not enough to prevent reactive oxygen species generation and vascular dysfunction in renovascular hypertension. Eur J Pharmacol 821:97–104. 10.1016/j.ejphar.2018.01.004 29331564 10.1016/j.ejphar.2018.01.004

[CR23] Massa CM, Liu Z, Taylor S et al (2021) Biological mechanisms of s-nitrosothiol formation and degradation: how is specificity of s-nitrosylation achieved? Antioxidants. 10.3390/antiox10071111 34356344 10.3390/antiox10071111 PMC8301044

[CR24] Mengel A, Chaki M, Shekariesfahlan A, Lindermayr C (2013) Effect of nitric oxide on gene transcription - S-nitrosylation of nuclear proteins. Front Plant Sci 4:293. 10.3389/fpls.2013.00293 23914201 10.3389/fpls.2013.00293 PMC3729996

[CR25] Miguel-Carrasco JL, Zambrano S, Blanca AJ et al (2010) Captopril reduces cardiac inflammatory markers in spontaneously hypertensive rats by inactivation of NF-kB. J Inflamm 7:1–9. 10.1186/1476-9255-7-21/FIGURES/4 10.1186/1476-9255-7-21 PMC2879251 20462420

[CR26] Moncada S, Palmer RMJ, Higgs Ea (1991) Nitric oxide: physiology, pathophysiology, and pharmacology. Pharmacol Rev 66:37–39 1852778

[CR27] Montenegro MF, Sundqvist ML, Larsen FJ et al (2017) Blood pressure-lowering effect of orally ingested nitrite is abolished by a proton pump inhibitor. Hypertension 69:23–31. 10.1161/HYPERTENSIONAHA.116.08081 27802417 10.1161/HYPERTENSIONAHA.116.08081

[CR28] Neto-Neves EM, Pinheiro LC, Nogueira RC et al (2019) Sodium nitrite improves hypertension-induced myocardial dysfunction by mechanisms involving cardiac S-nitrosylation. J Mol Cell Cardiol 134:40–50. 10.1016/j.yjmcc.2019.06.012 31226341 10.1016/j.yjmcc.2019.06.012

[CR29] Nott A, Watson PM, Robinson JD et al (2008) S-nitrosylation of histone deacetylase 2 induces chromatin remodelling in neurons. Nature 455:411–415. 10.1038/nature07238 18754010 10.1038/nature07238

[CR30] Oliveira-Paula GH, Batista IMR, Stransky S et al (2023) Orally administered sodium nitrite prevents the increased α-1 adrenergic vasoconstriction induced by hypertension and promotes the S-nitrosylation of calcium/calmodulin-dependent protein kinase II. Biochem Pharmacol. 10.1016/j.bcp.2023.115571 37127250 10.1016/j.bcp.2023.115571 PMC10198929

[CR31] Pechanova O, Kashiba M, Inoue M et al (1999) Role of glutathione in stabilization of nitric oxide during hypertension developed by inhibition of nitric oxide synthase in the rat. Jpn J Pharmacol 81:223–229 10591481 10.1254/jjp.81.223

[CR32] Pinheiro LC, Montenegro MF, Amaral JH et al (2012) Increase in gastric pH reduces hypotensive effect of oral sodium nitrite in rats. Free Radic Biol Med 53:701–709. 10.1016/j.freeradbiomed.2012.06.001 22721923 10.1016/j.freeradbiomed.2012.06.001

[CR33] Pinheiro LC, Amaral JH, Ferreira GC et al (2014) The antihypertensive effects of sodium nitrite are not associated with circulating angiotensin converting enzyme inhibition. Nitric Oxide 40:52–59. 10.1016/j.niox.2014.05.009 24878382 10.1016/j.niox.2014.05.009

[CR34] Pinheiro LC, Amaral JH, Ferreira GC et al (2015) Gastric S-nitrosothiol formation drives the antihypertensive effects of oral sodium nitrite and nitrate in a rat model of renovascular hypertension. Free Radic Biol Med 87:252–262. 10.1016/j.freeradbiomed.2015.06.038 26159506 10.1016/j.freeradbiomed.2015.06.038

[CR35] Pinheiro LC, Ferreira GCGC, Vilalva KHKH et al (2016) Oral nitrite circumvents antiseptic mouthwash-induced disruption of enterosalivary circuit of nitrate and promotes nitrosation and blood pressure lowering effect. Free Radic Biol Med 101:226–235. 10.1016/j.freeradbiomed.2016.10.013 27769921 10.1016/j.freeradbiomed.2016.10.013

[CR36] Pinheiro LC, Ferreira GCGC, Vilalva KHKH et al (2018) Contrasting effects of low versus high ascorbate doses on blood pressure responses to oral nitrite in L-NAME-induced hypertension. Nitric Oxide 74:65–73. 10.1016/j.niox.2018.01.006 29378249 10.1016/j.niox.2018.01.006

[CR37] Pinheiro LC, Oliveira-Paula GH, Ferreira GC et al (2021) Oral nitrite treatment increases S-nitrosylation of vascular protein kinase C and attenuates the responses to angiotensin II. Redox Biol. 10.1016/j.redox.2020.101769 33126056 10.1016/j.redox.2020.101769 PMC7596338

[CR38] Riccio A, Alvania RS, Lonze BE et al (2006) A nitric oxide signaling pathway controls CREB-mediated gene expression in neurons. Mol Cell 21:283–294. 10.1016/j.molcel.2005.12.006 16427017 10.1016/j.molcel.2005.12.006

[CR39] Rieder MJ, Carmona R, Krieger JE et al (1997) Suppression of angiotensin-converting enzyme expression and activity by shear stress. Circ Res 80:312–319. 10.1161/01.RES.80.3.312 9048650 10.1161/01.res.80.3.312

[CR40] Rizzi E, Guimaraes DA, Ceron CS et al (2014) β1-adrenergic blockers exert antioxidant effects, reduce matrix metalloproteinase activity, and improve renovascular hypertension-induced cardiac hypertrophy. Free Radic Biol Med 73:308–317. 10.1016/j.freeradbiomed.2014.05.024 24933619 10.1016/j.freeradbiomed.2014.05.024

[CR41] Sharifi AM, Akbarloo N, Heshmatian B, Ziai A (2003) Alteration of local ACE activity and vascular responsiveness during development of 2K1C renovascular hypertension. Pharmacol Res 47:201–209. 10.1016/S1043-6618(02)00319-5 12591015 10.1016/s1043-6618(02)00319-5

[CR42] Sharifi AM, Akbarloo N, Darabi R, Larijani B (2004) Study of correlation between elevation of blood pressure and tissue ACE activity during development of hypertension in 1K1C rats. Vascul Pharmacol 41:15–20. 10.1016/j.vph.2004.03.002 15135327 10.1016/j.vph.2004.03.002

[CR43] Sonoda K, Ohtake K, Uchida H et al (2017) Dietary nitrite supplementation attenuates cardiac remodeling in l -NAME-induced hypertensive rats. Nitric Oxide 67:1–9. 10.1016/j.niox.2017.04.009 28438687 10.1016/j.niox.2017.04.009

